# The Regulator of G Protein Signaling 14 Knockout Mouse, a Model of Healthful Longevity Protects Against Obesity and Glucose Intolerance Through a Brown Adipose Tissue Mechanism

**DOI:** 10.3390/ijms26094113

**Published:** 2025-04-26

**Authors:** Stephen F. Vatner, Jie Zhang, Marko Oydanich, Dorothy E. Vatner

**Affiliations:** Department of Cell Biology and Molecular Medicine, Rutgers, New Jersey Medical School, Newark, NJ 07103, USA

**Keywords:** RGS14, brown adipose tissue, obesity, glucose tolerance, longevity

## Abstract

The Regulator of G Protein Signaling 14 (*RGS14*) knockout (KO) mouse is a model of healthful longevity, i.e., its lifespan is prolonged and demonstrates enhanced exercise performance and protection against heart disease and hypertension. In this investigation, we found the RGS14 KO mouse is also protected against obesity and glucose intolerance by promoting a low white adipose tissue (WAT) phenotype with increased brown adipose tissue (BAT). This was confirmed by lower body weight, lower white adipocyte size, increased metabolism and improved glucose tolerance and insulin sensitivity. Upon examination of the white adipose tissue, RGS14 KO exhibited increased expression of “beiging” genes as well as significant increase in Uncoupling protein-1 (UCP-1) expression. The mechanism behind this protection was due to its unique brown adipose tissue. This was determined by BAT transplantation, which led to a reversal of phenotype, such that RGS14 BAT recipients developed protection similar to intact RGS14 KO mice, and the RGS14 KO BAT donors lost their protection. Thus, two novel mechanisms mediating obesity and glucose intolerance were found, i.e., inhibition of *RGS14* and its BAT.

## 1. Introduction

**Figure 1:**
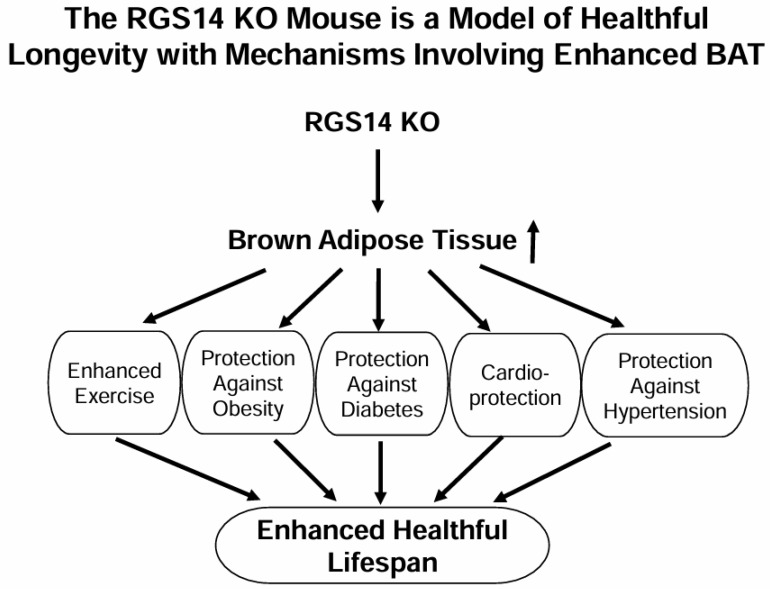
The Regulator of G Protein Signaling 14 (*RGS14*) knockout (KO) mouse is a model of healthful longevity with a mechanism involving enhanced brown adipose tissue (BAT). RGS14 KO mice exhibit cardio-protection and protection against obesity, diabetes, exercise intolerance and hypertension, mediated by its enhanced BAT.

Enhanced longevity in humans is a double-edged sword. On the one hand, longevity is considered beneficial. On the other, longevity can also be detrimental, by increasing incidence of heart disease, cancer, obesity, diabetes and exercise intolerance. It is therefore critical to find mechanisms to enhance healthful longevity, which would likely be derived from animal models of healthful longevity. One model, the mouse with the regulator of G protein signaling 14 disrupted (*RGS14* knockout (KO)) is likely to produce these new mechanisms, either through its genotype or secondarily to its brown adipose tissue (BAT). Our previous studies demonstrated that RGS14 KO mice exhibited extended lifespan, protection against cold exposure, improved thermogenesis and mitochondrial function through its unique BAT and upregulated SIRT3 [1]. In addition, RGS14 KO mice exhibited enhanced exercise capacity with improved anti-oxidant defense and blood flow/angiogenesis [2]. We have also found that BAT from this model of healthful longevity can recapitulate its salutary effects in mediating enhanced exercise capacity, metabolism, cardioprotection and lifespan and protection against hypertension (Figure 1) [1,2].

The goal of this investigation is to examine whether the RGS14 KO mouse protects against obesity and glucose intolerance, since obesity and diabetes can significantly impact life expectancy and quality of life [3,4]. Obesity can markedly reduce life expectancy, and the presence of diabetes can further the impact of obesity on lifespan [3]. First, we examined the role of the RGS14 KO genotype and secondly, we examined whether similar salutary effects could be achieved with the BAT from this mouse. The BAT from this mouse is more powerful than BAT from wild type (WT) mice, as reflected by its ability to increase exercise performance in WT littermates with RGS14 KO BAT transplantation in three days, as compared with eight weeks required when WT BAT was transplanted to other WT mice [2]. To accomplish these goals, we first examined baseline body weight, adiposity index, visceral fat pads, white adipose tissue staining and triglycerides in the RGS14 KO vs WT. We then examined the role of BAT mediating the positive effects of the RGS14 KO mice by repeating these experiments after removing BAT from RGS14 KO mice and transplanting it into WT and then examining the BAT donors and the BAT recipients. Next, we examined the effects of glucose tolerance using a glucose tolerance test and an insulin tolerance test and fasting blood glucose levels in the same four groups of mice, i.e., RGS14 KO, WT, WT with BAT transplanted from RGS14 KO mice (RGS14 KO BAT recipients) and RGS14 KO mice BAT donors.

## 2. Results

### 2.1. RGS14 Deficiency Improves Body Composition by Enhanced Brown Adipose Tissue

**Figure 2:**
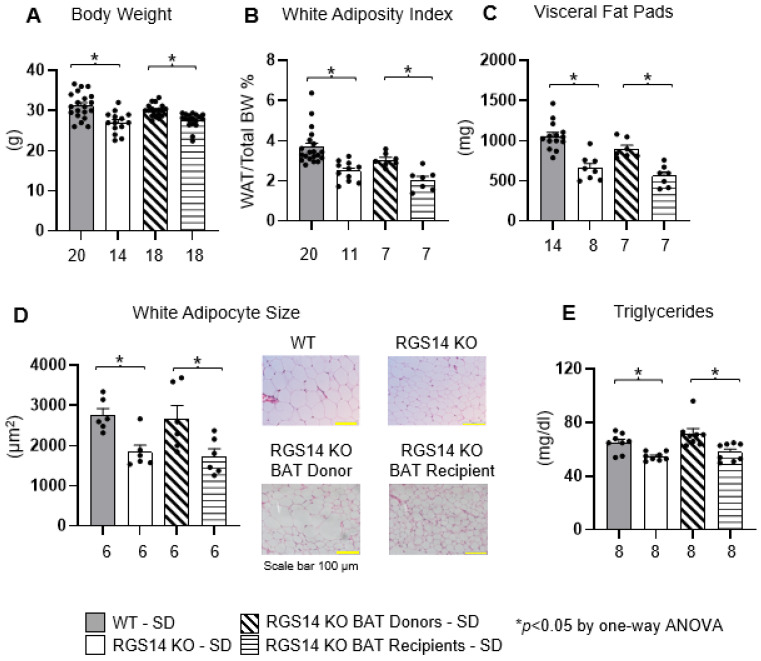
RGS14 KO mice consumed a similar amount of food, but body weight was lower (**A**), adiposity index was less (**B**), weight of visceral fat pads was lower (**C**), white adipocyte size was smaller with hematoxylin–eosin (H&E) staining (**D**) and triglycerides levels were lower (**E**), compared to WT littermates. After BAT transplantation, RGS14 KO BAT recipients exhibited similar trends to RGS14 KO mice, with lower body weight (**A**), less adiposity index (**B**), lower weight of visceral fat pads (**C**), smaller white adipocyte size (**D**) and lower triglycerides level (**E**), compared to RGS14 KO BAT donors. * *p* < 0.05 by one-way ANOVA with Tukey’s multiple comparisons test. X-axes represent the number of mice in the respective groups.

There were no significant differences in food intake among all four groups of mice, but body weight and white adipose tissue (WAT) adiposity index were lower in RGS14 KO mice than in WT littermates with standard diet (SD). After BAT transplantation, RGS14 KO BAT recipients exhibited improved body composition compared to RGS14 KO BAT donors, and similar to RGS14 KO mice, reflected significantly lower body weight (Figure 2A), lower WAT adiposity index (Figure 2B), lower visceral fat pad weight (Figure 2C), smaller WAT cell size (Figure 2D) and lower levels of triglycerides (Figure 2E).

**Figure 3:**
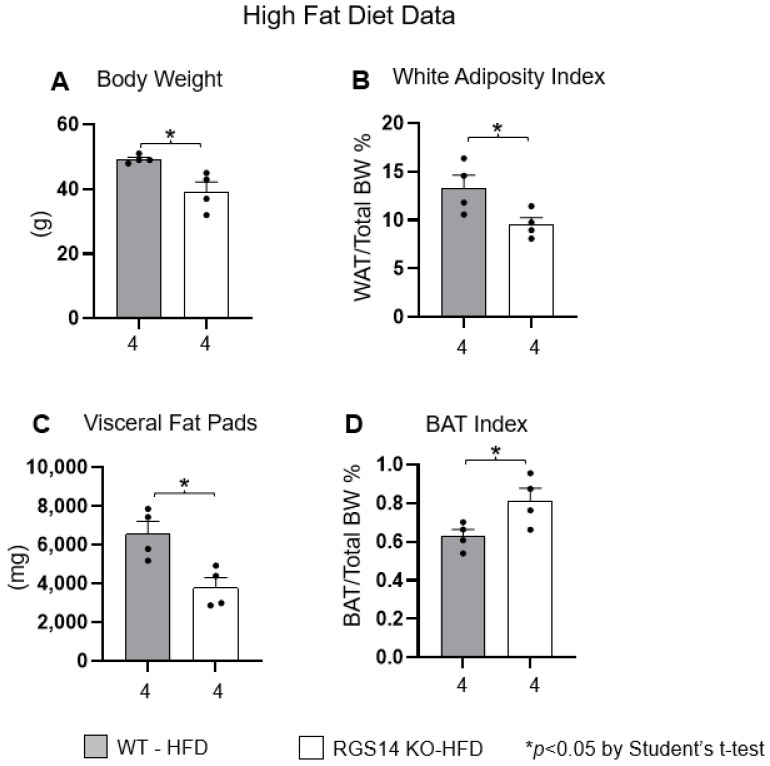
After 100 days on a HFD diet, RGS14 KO mice gained less weight (**A**), exhibited lower adiposity index (**B**), lower weight of visceral fat pads (**C**) and greater BAT index (**D**), compared to WT mice. * *p* < 0.05 by Student’s *t*-test for groups of 2. X-axes represent the number of mice in the respective groups.

With a high-fat diet (HFD) challenge for 100 days, the RGS14 KO mice exhibited lower body weight (Figure 3A), lower WAT adiposity index (Figure 3B), lower visceral fat pad weight (Figure 3C) and greater BAT index (Figure 3D).

### 2.2. RGS14 Deficiency Improves Glucose Tolerance and Insulin Sensitivity by Enhanced Brown Adipose Tissue

**Figure 4:**
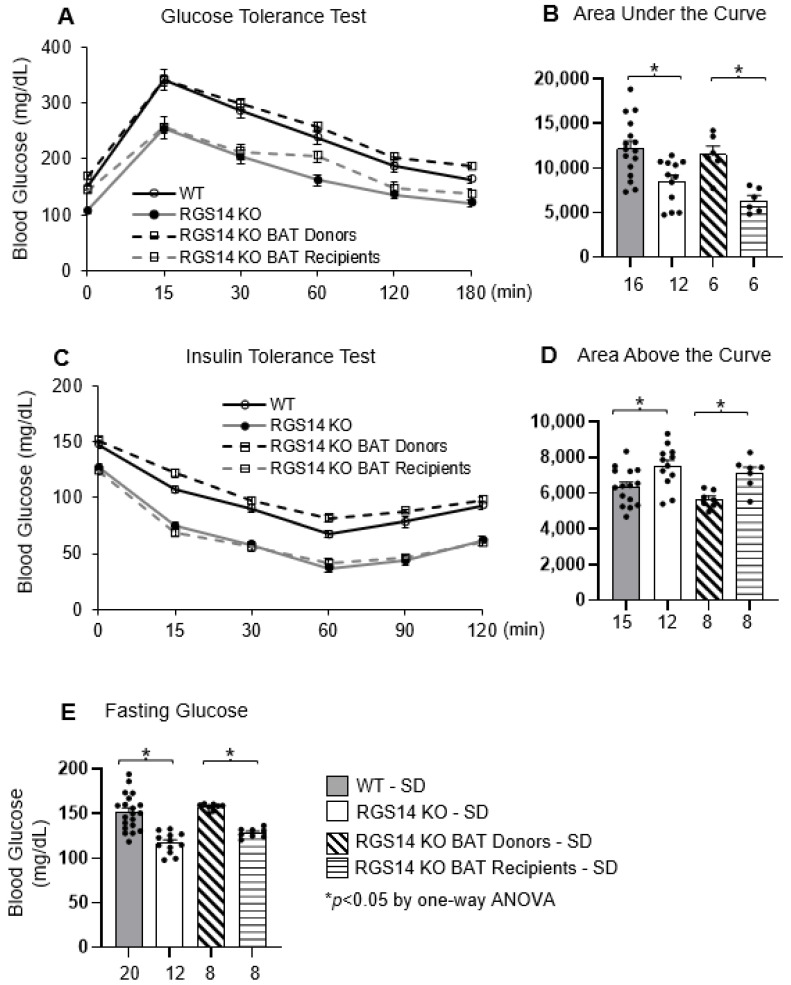
Compared with WT mice, RGS14 KO mice displayed improved glucose tolerance (**A**,**B**), enhanced insulin sensitivity (**C**,**D**) and reduced fasting glucose (**E**). After BAT transplantation, RGS14 KO BAT recipients exhibited similar trends as RGS14 KO mice, showing improved glucose tolerance shown as GTT curve (**A**) and GTT area under the curve (**B**), enhanced insulin sensitivity shown as ITT curve (**C**) and ITT area above the curve (**D**) and reduced fasting glucose (**E**), compared to RGS14 KO BAT donors. * *p* < 0.05 by one-way ANOVA with Tukey’s multiple comparisons test. X-axes represent the number of mice in the respective groups.

The RGS14 KO mice exhibited lower fasting glucose levels (Figure 4E) and an overall improvement in glucose tolerance (Figure 4A,B), measured as area under the curve, when compared to WT mice. This was due to increased insulin sensitivity in the RGS14 KO mice (Figure 4C,D).

After BAT was removed from RGS14 KO mice and transplanted to WT mice, the RGS14 KO BAT donors lost their enhanced glucose tolerance and insulin sensitivity, while their phenotype was transferred to the RGS14 KO BAT recipients (Figure 4).

### 2.3. RGS14 Deficiency Promotes White Adipose Tissue Browning by Enhanced Brown Adipose Tissue

**Figure 5:**
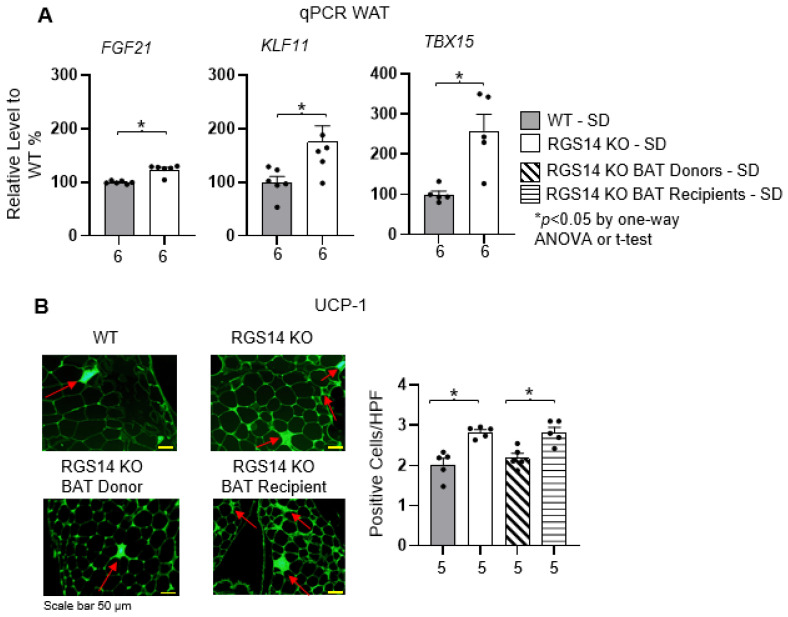
(**A**) RGS14 KO mice showed increased levels of genes related to WAT browning, determined by quantitative PCR of fibroblast growth factor 21 (FGF21:); Krüppel-like factor 11 (KLF11); T-box transcription factor 15 (TBX15). (**B**) By immunohistochemistry staining, RGS14 KO mice displayed greater levels of UCP1 in WAT, compared to WT mice, indicating WAT browning. RGS14 KO BAT recipient mice also showed higher levels of UCP1 in WAT compared to RGS14 KO BAT donors. The UCP1 staining was performed in fat from gonadal fat pads. The staining was done by fluorescence probes. Positive cells were determined within each sampled field (10–25 fields per sample). Example of positive cells are indicated by red arrows. * *p* < 0.05 by Student’s *t*-test for groups of two or one-way ANOVA with Tukey’s multiple comparisons test for groups of 4. X-axes represent the number of mice in the respective groups.

From RT-qPCR analysis to profile changes in WAT (gonadal fat) transcript levels, markers for WAT browning were significantly upregulated in RGS14 KO mice, when compared to WT mice (Figure 5A). Additionally, UCP-1, a key marker of WAT browning, was significantly increased in gonadal fat in RGS14 KO mice compared to WT mice, detected by immunofluorescence (Figure 5B). Similar to RGS14 KO mice, RGS14 KO BAT recipients also displayed a greater level of UCP-1 in WAT, compared to RGS14 KO BAT donors.

Overall, these data suggest that improved body composition, glucose tolerance and insulin sensitivity in RGS14 KO mice, and RGS14 KO BAT recipients were mediated by the unique BAT from the RGS14 KO mice.

## 3. Discussion

The major findings of this investigation demonstrated that the RGS14 KO mouse, a model of healthful longevity, has marked protection against obesity and glucose intolerance, two major factors that enhance healthful longevity. An additional major finding was that the BAT from the RGS14 KO mouse mediated these effects of protection against obesity and glucose intolerance, since removing BAT from the RGS14 KO mouse eliminated the protection, and when BAT was transplanted to WT mice, the protection against obesity and glucose intolerance was recapitulated in the WT mice recipients of the transplanted BAT. These factors of healthful longevity in the RGS14 KO mediated by BAT add to other factors demonstrated previously in our laboratory, i.e., extension of lifespan, enhanced exercise, metabolism, mitochondrial function, thermogenic function and antioxidant defense [1,2]. The current study is unique, compared with studies of mice with other RGS isoforms, as the marked protection of RGS14 KO mice against obesity and glucose intolerance was mediated by its BAT. In fact, some prior studies on other RGS isoforms found the opposite results [5,6,7,8,9].

BAT has been known for considerable time as enhanced in response to cold exposure, and with activation of the sympathetic nervous system to promote its thermogenic function [10]. This results in burning calories to produce heat, increasing energy expenditure, thereby preventing the accumulation of excess fat, and reducing obesity. White fat “browning” is where white fat cells develop characteristics similar to brown fat cells, particularly in the expression of UCP1. Consistent with this, Figure 5B shows UCP1 levels are higher in RGS14 KO mice and RGS14 KO BAT recipient mice. Browning agents promote the expression and activity of UCP1 in WAT, leading to increased heat production and energy expenditure [11]. A high-fat diet is linked to an increase in thermogenic capacity, elevated BAT mass and higher levels of UCP-1. Conversely, the ablation of UCP-1 results in reduced thermogenic capacity and increased susceptibility to diet-induced obesity [12]. BAT transplantation has recently been utilized to determine the relationship between BAT and obesity [13,14]. BAT also regulates lipid metabolism by decreasing triglycerides and cholesterol [13,14].

BAT also protects against glucose intolerance and insulin resistance [15,16] and against the pre-diabetic state [17], as well as allied metabolic disorders [18]. A recent human study reported that reduced BAT mass is associated with increased incidence of type 2 diabetes and cardiovascular disease [19]. Additionally, increasing BAT mass by transplantation improves glucose metabolism and insulin sensitivity in mice [13,20,21,22]. BAT transplantation also protects against both type 1 diabetes by improving glycemia with increased IGF-1 [23,24] and type 2 diabetes by improving glucose tolerance with increased IL-6 [13] or adiponectin [14].

Since diabetes and obesity significantly impact life expectancy and quality of life adversely, the findings that the RGS14 KO mouse and its BAT protect against obesity and glucose intolerance suggest novel mechanisms to be utilized clinically to combat the deleterious effects of obesity and diabetes in reducing healthful longevity.

## 4. Materials and Methods

### 4.1. Animal Experimental Procedures

All experiments were performed on 3–6 month old male RGS14 KO or RGS14 WT littermates. Two subgroups of mice, with BAT transplanted from RGS14 KO mice (BAT donors) to WT mice (BAT recipients) were also studied. The RGS14 KO mouse model was developed in our laboratory at Rutgers, New Jersey Medical School [1,2]. Animals were kept on a standard 12:12 h light–dark cycle and were all placed on standard chow and had free access to water for the duration of the study. Pups were weaned at 28 days of age and housed individually to allow for measurement of food intake on standard diet (SD) and bodyweight, in a pathogen-free facility.

RGS14 KO and WT mice were studied on SD and high fat diet (HFD), containing 60% of kcals from fat (BioServ, Frenchtown, NJ, USA) for 100 days as previously described [25]. Age- and sex-matched mice on SD were also followed as a control. These studies were approved by the Institutional Animal Care and Use Committee of Rutgers University, New Jersey Medical School.

### 4.2. Brown Adipose Tissue Removal and Transplantation

Three-to-six month old male RGS14 KO mice or WT littermates were anesthetized using pentobarbital (60 mg/kg). The backs of the mice were shaved, and the mice were placed in the prone position. A 2 cm midscapular transverse incision was made on the back of the mouse, and the BAT was freed from the surrounding muscles. Transplantation was initiated by removing BAT from the RGS14 KO mice (RGS14 KO BAT donor). The removed BAT from donor mice was incubated in 10 mL saline at 37 °C for 20–30 min. Three-to-six month old WT mice were anesthetized and 0.1 g of BAT from the RGS14 KO donor mice was subsequently transplanted into the visceral cavity of another WT mouse (RGS14 KO BAT recipient). The graft was carefully lodged deep between folds within the endogenous epididymal fat of the recipient mice. The skin incision in the recipient was closed with 6-0 nylon sutures. The skin incision in the donor was closed using stainless steel wound clips [1,2]. These mice were allowed to recover for at least 3 days prior to testing. Separate groups were used for the WT and BAT recipients and separate groups were used for comparing RGS14 KO mice with intact BAT and BAT donors after removing BAT. Animals were age and sex matched and randomized and tested blindly for the measurements listed below.

### 4.3. Fasting Glucose and Lipid Profile

After a 6 h fast, animals were anesthetized with 290 mg/kg i.p. Avertin, and the blood samples were drawn for fasting glucose and triglycerides. The glucose was measured with a glucometer (Accu-Check, Roche, Indianapolis, IN, USA). The triglycerides were measured using CardioChek PA Analyzer (PTS Diagnostics, Whitestown, IN, USA) [25].

### 4.4. Calculation of White Adiposity Index and BAT Index

Gonadal, perirenal, retroperitoneal and inguinal fat pads were isolated and weighed. The white adiposity index was calculated using total white adipose depot (gonadal, retroperitoneal, inguinal) weight divided against live body weight then multiplied by 100. The visceral fat pads weight is the total weight of perirenal, retroperitoneal and gonadal fat pads [25]. The brown fat adiposity index was calculated using brown adipose depot weight divided by live body weight then multiplied by 100 [1].

### 4.5. Glucose Tolerance Test

Mice were fasted for 6 h prior to initiation of the glucose tolerance test [25]. A 50 µL blood sample was drawn at the end of the fasting period for basal glucose measurement with a glucometer (Accu-Check, Roche, Indianapolis, IN, USA). A blood sample was collected from a venous tail puncture, and blood glucose was measured for basal glucose measurement with an Accu-Chek glucometer. A dose of dextrose (50% solution, 1 g/kg body weight) was injected intraperitoneally, and to calculate the glucose tolerance curve, blood was drawn at 15, 30, 60, 90, 120 and 180 min after injection for glucose measurements.

### 4.6. Insulin Tolerance Test

Similar to the glucose tolerance test, mice were fasted for 6 h prior to initiation of the insulin tolerance test [25]. A 50 µL blood sample was drawn at the end of the fasting period for basal glucose measurement with a glucometer (Accu-Check, Roche, Indianapolis, IN, USA). A dose of Humalog 1 U/kg (Eli Lilly, Indianapolis, IN, USA) was injected intraperitoneally, and to calculate the curve blood was drawn at 15, 30, 60, 90 and 120 min for blood glucose determination.

### 4.7. Quantitative RT-PCR

Specific primers and probes (derived with FAM and TAMRA, ordered from IDT DNA Company) were designed for the transcripts of interest. The optimal combination of primers and probes for a qPCR assay was determined with the Primer Express software (version 3.0, Applied Biosystems, Foster City, CA, USA). Following reverse transcription of the mRNA of interest from 50 ng of total RNA, the cDNA was used for quantitative PCR (qPCR) (40 cycles of a 10 s step at 95 °C and a 1 min step at 60 °C) using the SybrGreen method on a 7700 ABI-Prizm Sequence Detector (Applied Biosystems, Foster City, CA, USA). Values are reported per cyclophilin transcript to correct for sample-to-sample RNA loading variations.

### 4.8. Histology and Immunofluorescence Staining

Fat pads were collected from the mice, which were fixed with 10% formalin. Paraffin-embedded samples were sectioned at 5 µm thickness. The BAT and visceral fat pads, which include perirenal, retroperitoneal and gonadal fat pads, were stained with hematoxylin–eosin (H&E) [25]. Adipocyte cell size was measured and counted in ImageJ (version 1.53i) using digital calipers at 100× magnification.

UCP-1 positive cells were stained by UCP-1 antibody (ab209483, Abcam, Waltham, MA, USA) and were quantified at 200× magnification as absolute number per unit in gonadal fat pads. Positive cells were determined within each sampled field (10–25 fields per sample).

### 4.9. Statistical Analyses

All data are expressed as mean ± SEM. To compare two independent groups, we used the Student’s unpaired *t*-test, whereas for more than two groups, one-way analysis of variance (ANOVA) with Tukey’s multiple comparisons test was used. *p* < 0.05 was taken as the level of significance.

## Data Availability

Data will be made available upon reasonable request.
